# Neuron-derived neurotrophic factor promotes the differentiation of intramuscular and subcutaneous adipocytes in goat

**DOI:** 10.1080/10495398.2024.2346223

**Published:** 2024-05-13

**Authors:** An Li, Youli Wang, Ruiwen Li, Yaqiu Lin, Yanyan Li, Yong Wang, Wei Liu, Xiong Yan

**Affiliations:** a College of Animal and Veterinary Sciences, Southwest Minzu University, Chengdu, China; b Key Laboratory of Qinghai-Tibetan Plateau Animal Genetic Resource Reservation and Utilization, Ministry of Education, Southwest Minzu University, Chengdu, China; c Key Laboratory of Sichuan Province for Qinghai-Tibetan Plateau Animal Genetic Resource Reservation and Exploitation, Southwest Minzu University, Chengdu, China; d Chengdu Women’s and Children’s Central Hospital, School of Medicine, University of Electronic Science and Technology of China, Chengdu, China

**Keywords:** *NENF*, intramuscular adipocyte, subcutaneous adipocyte, adipocytes differentiation

## Abstract

Adipocyte play an important role in human health and meat quality by influencing the tenderness, flavor, and juiciness of mutton It has been shown that neuron-derived neurotrophic factor (*NENF*) is closely related to energy metabolism and adipocyte differentiation in bovine. However, the role of *NENF* in the goats remains unclear. The aim of this study was to detect the expression of *NENF* in goat subcutaneous and intramuscular adipocytes, temporal expression profiles of the *NENF*, and overexpressed *NENF* on the differentiation of different adipocytes. In this study, PCR amplification successfully cloned the goat *NENF* gene with a fragment length of 521 bp. In addition, the time point of highest expression of *NENF* differed between these two adipocytes differentiation processes. Overexpression of NENF in intramuscular and subcutaneous adipocytes promoted the expression levels of differentiation markers *CEBPβ* and *SREBP*, which in turn promoted the differentiation of intramuscular and subcutaneous adipocytes. This study will provide basic data for further study of the role of goats in goat adipocyte differentiation and for the final elucidation of its molecular mechanisms in regulating goat adipocyte deposition.

## Introduction

With the development of the Chinese economy, mutton consumption has risen.^1^ Adipocyte play a critical role in meat quality by affecting the tenderness, flavor, and juiciness of mutton.^2^ Analyzing the mechanisms of adipose tissue deposition helps breeders to regulate lipid accumulation at the molecular level, thereby improving meat quality. Lipid deposition is primarily dependent on proliferation and differentiation of adipocytes, and a variety of genes and transcription factors are involved in these processes.^3^^,^^4^

Neuron-derived neurotrophic factor (*NENF*), originally named neudesin, belongs to the membrane-associated progesterone receptor (MAPR) protein family known as candidate cancer genes, is a new type of secretory protein found in mouse embryos,^5^^,^^6^ which is involved in nervous system development, energy metabolism, and tumorigenesis.^7–9^ *NENF* is widely expressed in brain, heart, lungs, kidneys, spinal cord, and embryo.^10^ The neurotrophic activity of recombinant *NENF* combined with exogenous heme (NENF-hemin) in primary cultured neurons and Neuro2a cells was significantly higher than that of recombinant *NENF*, showing that the activity of NENF depends on hemin.^11^ In addition, *NENF* is a important central regulator of food intake,^12^ and also functions in maintaining the hippocampal anxiety circuit. Recent studies in cattle have shown that *NENF* plays an important role in adipocyte differentiation, and deletion of *NENF* inhibits the differentiation of preadipocytes and promotes myogenesis of myoblasts.^13^ In addition, *NENF* suppresses adipogenesis in 3T3-L1 cells via the MAPK cascade.^10^

Current research on *NENF* has focused on animals, such as mice and cattle. However, few studies have been conducted in goats, and studies of adipocytes have seldom been reported. Therefore, in this study, tissues and cells from Jianzhou Big-eared goats were used to explore the role of *NENF* in adipogenesis. Fat deposition is the key factor affecting the meat quality traits of goats, which could be improved by genetic manipulation of fat deposition-related genes, which provides a basic theory for improving goat meat quality and breeding.

## Materials and methods

### Cloning and sequence analysis

Jianzhou Big-eared goats are a new breed of goats in China: a new breed of meat goats crossed between African native Nubian goats and Jianyang local goats,^14^ which has the advantages of fast growth rate, excellent meat production performance, high reproductive performance, large size, stable genetic performance, tolerance to rough breeding, and adaptation to the subtropical climate conditions of southern China. We selected one-year-old Jianzhou Big-eared goats as experimental animals. According to the predicted sequence of Bos indicus with accession the number (XM 019976852.1). A specific pair of primers was designed using primer primers 5.0 software. PCR system: 22 μL T_3_ Mix (Tiangen, Beijing, China), 1 μL template (1 μg/μL), and upstream and downstream primers at a concentration of 10 μmol/L. The PCR procedure: pre-degeneration (94 °C, 4 min), degeneration (94 °C 30 s), annealing (58 °C 15 s), extension (72 °C, 90 s) total for 38 cycles. Subsequently, 1% agarose gel electrophoresis was used to detect the amplified products, and the target fragments were recovered using a DNA purification Kit. The product was ligated with 007VS vector (Tsinke, Beijing, China) and transformed into trelief® ^5α^ Chemically Competent Cell. Colonies with positive were picked on Amp + plates. The were analyzed using bacterial liquid PCR. Finally, it was then sent to Chengdu SanGon Biotechnology Co. Ltd for sequencing. Tools of bioinformatics analysis of goat NENF were shown in Table 1.

**Table 1.** The tools for analysis and the content of their analysis are listed in the table.

Software or online tools	Analytical content
Primer Primiers 5.0	Primer design
ORF Finder	Open reading frame prediction and amino acid sequence translation
ExPASy ProtParam	Analysis of physicochemical properties of protein
ExPASy	Prediction of protein secondary structure
SWISS-MODEL	Prediction of protein tertiary structure
SignalP4.1 Server	Signal peptide analysis
TMHMM	Prediction of transmembrane domain
PSORTII	Subcellular localization
STRING	Protein interaction analysis
MEGA5.0	Construction of phylogenetic tree
Blast	Homology comparison analysis

### Construction of recombinant plasmid of NENF overexpression

Based on the NENF sequence of Jianzhou Big-earedgoats obtained from the above cloning, subclone primers were designed according to their CDS region sequences. OE-NENF sense primer: CCGGAATTCCATGGCAGTCAAGGGGGTG; OE-NENF antisense primer: CCGCTCGAGACTCAGAACTCATCCTTTATGTCG. We use the cloned plasmid as a template for PCR amplification by subclone primers and purification for recovery.The PCR product was connected to the plasmid PEGFP-N1 and dual digestion (restriction sites *ECOR* I and *BAMH* I) was performed, followed by overnight ligation by T_4_ ligase at 16 °C. After resolving to DH5_α_ sensor cells, positive colonies were selected and sequenced.

### Sampling and cell culture

All experiments complied with the requirements of the Directory of Ethical Treatment of Experimental Animals of China. In addition, the experimental procedures were approved by the Institutional Animal Care and Use Committee, Southwest Minzu University (2020086, 2020).

The one-year-old male Jianzhou Big-eared goat (n = 3) purchased from Sichuan Jian yang Tiandi Animal Husbandry Co., Ltd. Goats were slaughtered, and tissues, including heart, liver, spleen, kidney, subcutaneous fat, longissimus dorsi, perirenal and lung were quickly collected and stored in liquid nitrogen.

Intramuscular adipocytes and subcutaneous adipocytes were taken from longissimus dorsi and abdomen of one-year-old male Jianzhou Big-eared goat(n = 3), respectively, washed twice in phosphate-buffered saline supplemented with 1% penicillin/streptomycin and then minced under sterile conditions. Enzymatic digestion was performed with 0.2% collagenase type II (Sigma, Germany) at 37 °C for 1h with gentle shaking and terminated with an equal volume of DMEM/F 12 (HyClone, Logan, USA) supplemented with 10% fetal bovine serum (FBS). Then the digestion solution was then centrifuged at 2000 rpm for 3 min. After staining, cells containing lipid droplets were stained red, and other cell types without lipid droplets cannot be stained, thus identifying the cells. The cell purity in this study reaches more than 75%.^15^ After discarding the supernatant, the cells were transferred to a culture flask to complete recovery. When the confluence reached 80%, the cells were suspended with trypsin (HyClone, Logan, USA) centrifuged at 2000 rpm for 3 min, and subsequently resuspended thoroughly using a culture medium. The cells were adjusted to a density of 1 × 10^6^/mL using counting plates and seeded into a culture bottle or dish. After the third passage (F3), cells were grown to 80% confluence, 50 μmol/L of oleic acid (Sigma, Germany) was added to the culture medium to induce preadipocyte adipogenesis differentiation, as previously reported.^16^

### Detection of NENF overexpression efficiency and its effect on subcutaneous and intramuscular adipocytes lipid accumulation

When the density of goat intramuscular and subcutaneous preadipocytes reached 80%, each well was transfected with 1000 ng of empty PEGFP-N1 or PEGFP-N1-NENF plasmid mixed with 400 μL of Opti-MEM and 3 μL of transfection reagent (Turbofect, Thermo, Massachusetts, USA). Cells were cultured in an incubator at 37 °C and 5% CO_2_. Finally, the transfection solution was removed after 18 h and 50 μmol/L oleic acid was used to induce cell differentiation for 48 h.

Oil Red O staining was used to detect changes in lipid content in subcutaneous and intramuscular adipocytes after transfection with the overexpression vector. The Oil Red O solution was added to distilled water to prepare the Oil Red O staining solution, and the subcutaneous and intramuscular adipocytes were fixed with 10% formaldehyde solution for 30 min. The formaldehyde solution was then removed, soaked in 500 μL of Oil Red O staining, and washed with phosphate-buffered saline after staining. The lipid contents of the subcutaneous and intramuscular adipocytes of the goats were scanned under a microscope. Finally, lipids were extracted with 200 μL isopropanol and the absorbance was measured at 492 nm.

### Quantitative polymerase chain reaction and data processing and analysis

We selected a universally expressed transcription gene (UXT: XM_005700842.2) as the internal reference gene for normalization. The primer concentration and reaction system were the same as 1.1; qPCR reaction procedure consists of four steps consisting of pre-denaturation 95 °C, 3 min, degradation 95 °C, 10 s, annealing 58 °C, 10 s, and extension 72 °C, 15 s, of which degeneration, annealing, and extension were running for 38 cycles. The qPCR results were analyzed using the 2^-ΔΔCt^ method. All data in this experiment were shown as means ± SEM and analysis of variance in SPSS software was used to compare significance one-way ANOVA. Tukey’s test was used for multiple comparisons. Differences were considered statistically significant at *P* < 0.05. All experiments were repeated three times. The gene primer information is shown in Table 2.

**Table 2.** Information of primers.

Target Gene	Primer sequence	Application	Accession	Length(bp)
NENF	S:GAGCCCATCTACATGGCAGTC A:GTAATTCCACTGCCCACGAA	Cloning	XM_018060749.1	
NENF	S: GCAGACCTCACCCATGACA A: GAGGATTCTTCGGGCTGTAT	qPCR	XM_018060749.1	123
AP2	S: GTCCTTCAAATTGGGCCAGGA A: CTGGTGGTAGTGACACCGTT	qPCR	NM_001285623.1	192
CEBPα	S:CCGTGGACAAGAACAGCAAC A:AGGCGGTCATTGTCACTGGT	qPCR	XM_018062278.1	142
CEBPβ	S: CAAGAAGACGGTGGACAAGC A: AACAAGTTCCGCAGGGTG	qPCR	XM_018058020.1	204
SREBP	S: AAGTGGTGGGCCTCTCTGA A: GCAGGGGTTTCTCGGACT	qPCR	NM_001285755.1	127
PREF-1	S: CCTGAAAATGGATTCTGCGACG A: GACACAGGAGCACTCGTACTG	qPCR	NM_001314212.1	255
LPL	S: TCCTGGAGTGACGGAATCTGT A: GACAGCCAGTCCACCACGAT	qPCR	NM_001285607.1	174
PPARγ	S: AAGCGTCAGGGTTCCACTATG A: GAACCTGATGGCGTTATGAGAC	qPCR	NM_001285658.1	197
ADRP	S: TACGATGATACAGATGAATCCCAC A: CAGCATTGCGAAGCACAGAGT	qPCR	>NM_001285596.1	202
ATGL	S: CAAGGAGACGACGTGGAACA A: CATAGATGTGCGTGGCGTTG	qPCR	XM_018042656.1	125
DGAT1	S: CCACTGGGACCTGAGGTGTC A: GCATCACCACACACCAATTCA	qPCR	XM_018058728.1	101
GPAM	S: CCAGTATCCCGTCTTTGGGT A: TTACGTTGGTGGCAAACATGC	qPCR	XM_013975269.2	147
ACC	GGAGACAAACAGGGACCATT ATCAGGGACTGCCGAAAC	qPCR	XM_018064174.1	146
UXT	S: GCAAGTGGATTTGGGCTGTAAC A: ATGGAGTCCTTGGTGAGGTTGT	qPCR	XM_005700842.2	180

## Results

### Cloning and sequence analysis of NENF in Jian Zhou Da-er goat

**Figure 1.**
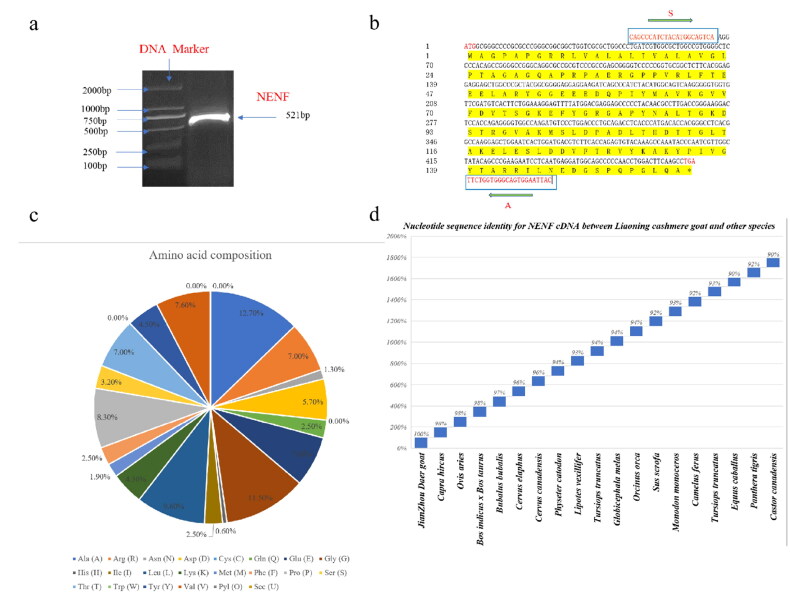
Cloning and sequence analysis of *NENF*. (a) Amplification of *NENF* gene in Jianzhou Big-eared goat; DNA marker DL2000, *NENF* target skip. (b) The sequences of nucleotide and deduced amino acid of NENF cDNA in Jianzhou Big-eared goat. The two green arrows indicated the positions of primer pair. The start codon ATG and stop codon TGA are represented by red bases, respectively. (c) The composition of deduced amino acid for NENF protein in Jianzhou Big-eared goat. (d) Nucleotide sequence identity of NENF cDNA between Jianzhou Big-eared goats and other mammalian species.

Using the *NENF* primers (NENF-S, NENF-A) and goat muscle cDNA as a template, PCR amplification successfully cloned the goat *NENF* gene. The fragment length was 521 bp (Fig. 1a), with a CDS region was 474 bp with the start codon ATG and stop codon TGA (Fig. 1a), and the open reading frame was 474 bp, encoding 158 amino acids (Fig. 1b).

**Figure 2.**
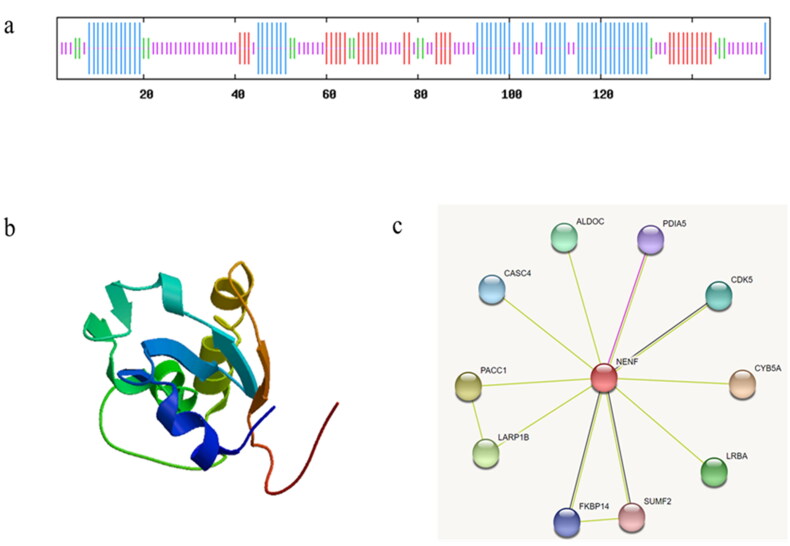
Structural analysis of NENF protein in goat. (a) Secondary structure prediction of NENF protein, α-helix are represented in blue, extended strand was represented in red, β-turn are represented in green, random coils are represented in orange. (b) Prediction of tertiary structure of NENF protein. (c) NENF protein interaction network.

We analyzed the amino acid sequence of goat *NENF* by the online tool (ExPASY), and the predicted protein molecular formula was C_741_H_1181_N_205_O_227_S_3_, and the *NENF* cDNA sequence encoded peptides with a length of 158 amino acid residues, the amino acid composition was shown in Fig. 2c. Fig. 2d shows the nucleotide sequence similarities of *NENF* between Jianzhou Big-eared goats and other mammalian species retrieved from GenBank. The nucleotide sequences obtained by the Jianzhou Big-eared had 90–98% agreement in *NENF* ORF cDNA compared to the corresponding sequences in the GenBank database of other mammalian species (Fig. 2d). These results suggest that *NENF* is highly evolutionarily conserved among different species.

The predicted molecular weight of the *NENF* protein was 16689.95 daltons and the isoelectric point (IP) was 5.48. Twenty negatively charged residues (Asp + Glu) and eighty positively charged residues (Arg + Lys) indicated that the protein encoded by *NENF* may be negatively charged. The instability index (II) was calculated as 53.53 and the hydrophilic large mean was 0.264, which means that *NENF* in goats is a hydrophilic unstable protein. In addition, we found that the goat *NENF* protein had no transmembrane domain and no signal peptides. Subcellular localization indicated that it was predominantly present in the cytoplasm (52.2%), followed by the nucleus (17.4%), and mitochondria (8.7%). The evolutionary process of goat *NENF* protein was studied by constructing a phylogenetic tree by MEGA 5.0 (Figure S1). The results showed that the Jianzhou Big-eared and Bos indicus × Bos taurus belonged to one class, and the relationship was closer, because they were in the same branch as bovine ruminants, the furthest away from the Globicephala melas, which is in line with the evolutionary laws of the species.

### *Structural analysis of* NENF *protein in goat*

Predictive analysis of the secondary structure showed that the NENF protein contained 52 α-helices (33.12%), 30 extended strands (19.11%), 13 β-turns (8.28%), and 62 random coils (39.49%, Fig. 2a). The predicted tertiary structure is similar to that of the secondary structure (Fig. 2b). From the interaction network analysis, we observed that the goat NENF protein may interact with a variety of proteins, such as CYB5A, CDK5, PDIA5, ALDOC, CASC4, PACC1, LARP1B, FKBP14, SUMF2, and LRBA.

### Relative expression levels of NENF in goat intramuscular and subcutaneous adipocytes

**Figure 3.**
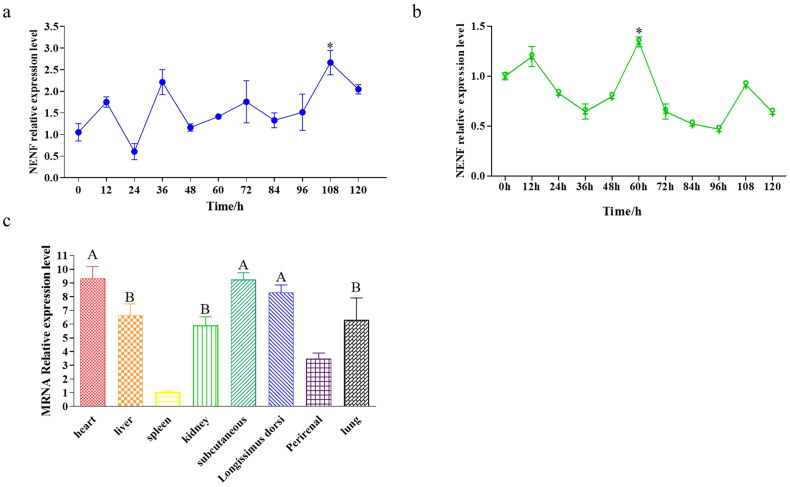
Relative expression level of *NENF* in goat tissues and subcutanesous adipocytes and intramuscular adipocytes. (a) Relative expression of *NENF* during differentiation of intramuscular adipocytes. (b) Relative expression of *NENF* during differentiation of subcutaneous adipocytes. *significant difference. (c) Relative expression level of *NENF* in different tissues in goat. ‘A’ means extremely significant difference, ‘B’ means significant difference.

After induction of differentiation, we harvest cells at 0, 12, 24, 36, 48, 60, 72, 84, 96, 108, and 120 h. Relative expression levels of *NENF* at different stages of cells were detected by qPCR. We used 0 h expression level as the control, and the results showed that the relative expression level of *NENF* during the differentiation of intramuscular preadipocytes was the highest at 108 h and significantly higher than that at 0 h (*P* < 0.05, Fig. 3a). In the temporal expression of subcutaneous preadipocytes, the relative expression level of this gene was highest at 60 h, which was significantly higher than that of 0 h (*P* < 0.05, Fig. 3b). Subsequently, *NENF* expression was detected in the heart, liver, spleen, kidney, subcutaneous tissue, longissimus dorsi, perirenal tissue, and lung. The results showed that *NENF* was widely expressed in goat tissues, except for the spleen and perirenal (Fig. 3c). The highest level of *NENF* was in subcutaneous adipose tissue (Fig. 3c). Using perirenal tissue as a control, *NENF* expression was significantly higher in heart, subcutaneous, and longissimus dorsi than in other tissues (*P* < 0.01), followed by higher levels in liver, kidney, and lung tissues than in other tissues tested with other selected samples (*P* < 0.05, Fig. 3c).

### NENF overexpression promotes goat adipocytes differentiation

**Figure 4.**
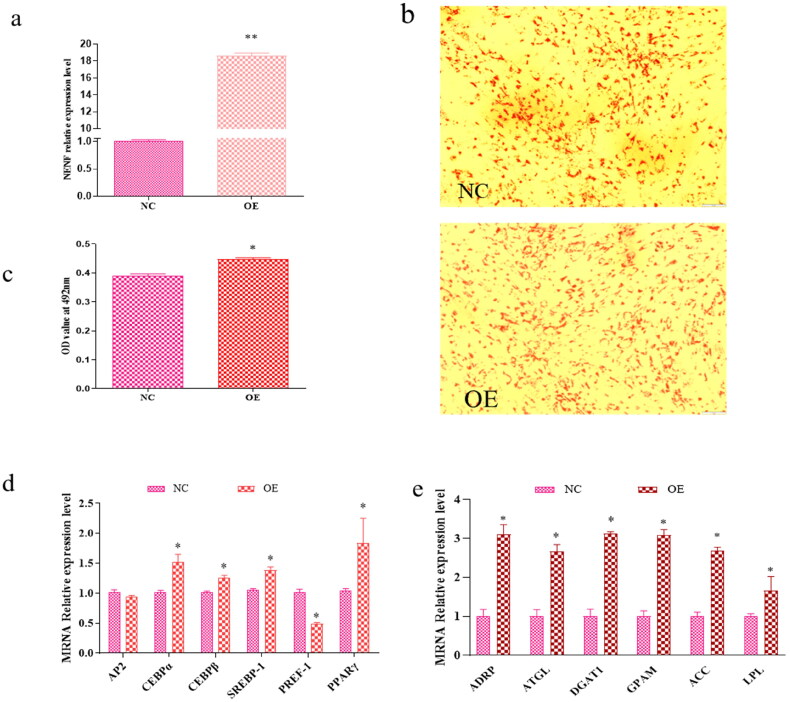
*NENF* overexpression promotes intramuscular adipocytes differentiation. (a) Quantitative polymerase chain reaction (qPCR) detects the overexpression efficiency of *NENF* in goat intramuscular adipocytes. (b) Oil red O staining of goat adipocytes. (c) Quantitative analysis of oil red O staining signal was indicated by absorbance at 492 nm. (d) Effect of overexpression of *NENF* on gene related to adipocytes differentiation of goat, expression changes in *AP2*, *CEBPα*, *CEBPβ*, *SREBP-1*, *PREF-1*, and *PPARγ* in intramuscular adipocytes. (e) Effects of *NENF* overexpression on genes associated with lipid synthesis in goat adipocytes. Changes in the expression of *ADRP*, *ATGL*, *DGAT1*, *GPAM*, *ACC*, and *LPL* in intramuscular adipocytes. *means *P* < 0.05, significant difference. **means *P* < 0.01, extremely significant difference.

**Figure 5.**
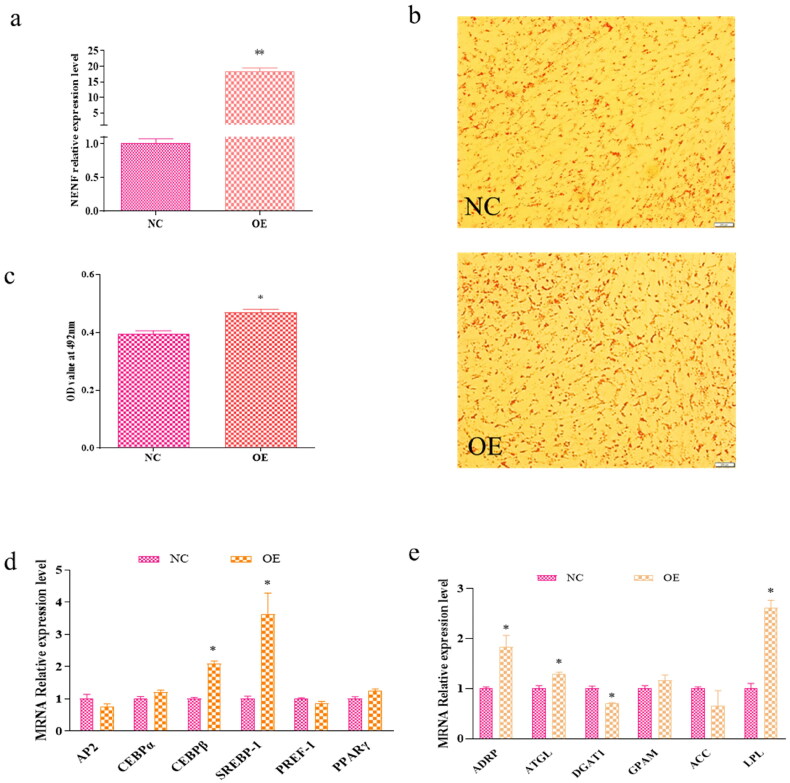
*NENF* overexpression promotes subcutaneous adipocytes differentiation. (a) qPCR detects the overexpression efficiency of *NENF* in goat subcutaneous adipocytes. (b) Oil red O staining of goat adipocytes. (c) Quantitative analysis of oil red O staining signal was indicated by absorbance at 492 nm. (d) Effect of overexpression of *NENF* on gene related to adipocytes differentiation of goat, expression changes in *AP2*, *CEBPα*, *CEBPβ*, *SREBP-1*, *PREF-1*, and *PPARγ* in subcutaneous adipocytes. (e) Effects of *NENF* overexpression on genes associated with lipid synthesis in goat adipocytes. Changes in the expression of *ADRP*, *ATGL*, *DGAT1*, *GPAM*, *ACC*, and *LPL* in subcutaneous adipocytes. *means *P* < 0.05, significant difference. **means *P* < 0.01, extremely significant difference.

To elucidate the exact role of *NENF* in adipogenesis, the functional gain of *NENF* was determined by transfection of OE-*NENF* overexpressed plasmids into primary cultured intramuscular and subcutaneous adipocytes. The result showed that overexpression of *NENF* was effectively detected as a 19-fold change in the intramuscular adipocyte group and a 16-fold change in the subcutaneous adipocyte group compared to the control group (Figs. 4a and 5a). In addition, increased lipid droplet signaling was observed in the overexpressed *NENF* group (Figs. 4b and 5b). Staining of OE plasmids and control-treated adipocytes with Oil Red O 2 days after induction of differentiation (Figs. 4b and 5b). Statistically, the OD value of the Oil red O signal in the overexpression group was significantly higher than that in the control group (*P* < 0. 05; Figs. 4c and 5c). The results showed that the overexpression of NENF in intramuscular adipocytes increased the expression levels of differentiation marker genes *CEBPα*, *SREBP*-1 and *PPARγ* and decreased the expression level of PREF-1. The expression levels of the lipid metabolism genes *ADRP*, *ATGL*, *DGAT*1, *GPAM*, *ACC*, and *LPL* were increased (Figs. 4d,e). On the other hand, the overexpression of *NENF* in subcutaneous adipocytes upregulated the expression levels of differentiation marker genes *SREBP-1* and lipid synthesis-related genes *ADRP*, *ATGL*, *LPL*, and *DGAT*1 (Fig. 5d,e). Thus, overexpression of *NENF* promotes adipocyte differentiation and lipid droplet accumulation.

### Comparison of the effects of NENF on the differentiation of goat intramuscular and subcutaneous adipocytes

To compare the effects of *NENF* on intramuscular and subcutaneous adipocyte differentiation in goats, we analyzed the differential expression of differentiation marker genes and lipid synthesis-related genes. Overexpression of *NENF* promotes goat intramuscular and subcutaneous adipocyte differentiation, and there are differences in the expression of genes that regulate differentiation markers. In general, the differentiation marker genes in both the intramuscular and subcutaneous adipocyte groups after overexpression of *NENF* showed the same tendency (Figs. 4d and 5d), by upregulating the expression of *C/EBPβ* and *SREBP-1*. Notably, the regulation of *NENF* in intramuscular adipocytes also involves upregulation of *CEBPα* and *PPARγ* and downregulation of *PREF-1*. For genes related to lipid synthesis, the expression levels of the lipid synthesis-related genes were all upregulated in intramuscular adipocytes, but in the subcutaneous adipocytes group, *DGAT1* showed a downward trend, and the expression of *GPAM* and *ACC* was not affected (Figs. 4e and 5e). These results suggest that the overexpression of *NENF* promotes the differentiation of intramuscular and subcutaneous adipocytes, but the degree of influence on the expression of related marker genes varies.

## Discussion

*NENF* is a secreted protein essential for a variety of biological processes. For instance, *NENF* is involved in protease-mediated neural differentiation in neural precursor cells,^17^ and its ectopic expression in macrophages cytolytic factor cells promotes tumor incidence.^18^ Moreover, deletion of *NENF* in mice leads to an increase in energy expenditure and heat production.^9^ Recently, *NENF* was reported to promote the differentiation of bovine preadipocytes.^10^ In this study, we explored the function of *NENF* in adipogenesis in goats.

In this study, we cloned the *NENF* gene in goats and predicted the genetic conservation of amino acids. The *NENF* gene is fully expressed in subcutaneous adipose tissue, and the highest mRNA expression level varies at different time points in the subcutaneous and intramuscular adipogenesis process. The sequence of the NENF protein was confirmed by interspecific phylogenetic tree comparison, and the homology was higher in ruminants (bovine) than in other species, suggesting that NENF plays a more conserved role in ruminants. The results of the NENF protein interaction analysis in goats showed that the NENF protein in goats may be interrelated with FKBP14, LARP1B, CASC4, ALDOC, PDIA5, CDK5*, and* CYB5A*. LARP1B*, *PDIA5* and *CDK5* are closely related to the occurrence of tumors and cancers, indicating that *NENF* and *LARP1B, PDIA5* and *CDK5* have antagonistic or synergistic effects in the development of obesity,^19–21^ *CASC4* causes breast cancer to occur,^22^ *ALDOC* plays an important role in the decomposition of carbohydrates during fat hydrolysis, which suggests that *ALDOC* and *NENF* may have antagonistic effects in adipogenesis,^23^ and *CYB5A* is closely associated with meat flavor.^24^ These genes may interact with *NENF* for various biological activities. Our study found that *NENF* had a wide range of expression levels. The characteristics and relatively high levels of expression in goat adipose tissues (liver and skin) were consistent with the expression in cattle.^8^ In addition, the time point of highest expression of *NENF* differed between these two adipocyte differentiation processes.

Aipocyte differentiation affects cell structure and function^25^ and is regulated by many transcription factors such as *AP2*, *CEBPα*, *CEBPβ*, *SREBP-1*, *PREF-1* and *PPARγ.*^26–29^ Among these, *SREBP-1* promote adipocyte differentiation by activating *PPARγ* expression.^27^^,^^28^ In this study, overexpression of *NENF* in goat intramuscular and subcutaneous adipocytes caused a similar change in adipocyte differentiation markers, but with significant differences. The results showed that the overexpression of *NENF* in intramuscular adipocytes promoted the expression levels of the differentiation marker genes *CEBPβ, CEBPα*, *SREBP-1* and *PPARγ*, while inhibiting the expression of *SREBP-1* in subcutaneous adipocytes. This is contrary to a previous study (Su et al., 2019), which showed that interfering with *NENF* reduced the expression levels of *PPARγ*, *CEBPα*, and *CEBPβ.*^13^ However, in subcutaneous adipocytes, the expression of *CEBPβ* and *SREBP-1* genes is promoted. In this regard, we deduced that *NENF* mainly promotes adipocyte differentiation through the expression of *CEBPβ, SREBP-1 and PPARγ* genes in goats.

Important genes, such as *ADRP*, *ATGL*, *DGAT*, *GPAM*, *ACC*, and *LPL* are closely associated with lipids.^30–34^ *ADRP* regulates the production of lipid droplets and lipids, *ATGL* overexpression reduces the triglyceride content, and LPL hydrolyzes low-density lipoprotein (*LDL*) to glycerol.^35^ As a rate-limiting enzyme for triglyceride synthesis, *DGAT* plays an important role in triglyceride synthesis.^36^ In this study, overexpression of *NENF* promoted the expression of genes related to triglyceride synthesis in intramuscular adipocytes, but there were differences in subcutaneous adipocytes, and it is worth noting that the expression of *DGAT1* was inhibited. Similarly, studies have shown that *NENF* promotes differentiation of bovine precursor adipocytes. In addition, *NENF* has different effects on different cell types, such as inhibition of myoblast differentiation.^37^ From these results, we can speculate that *NENF* promotes triglyceride synthesis. This study clarifies the key role of *NENF* as a positive regulator of goat adipocyte differentiation and lays the foundation for subsequent research on *NENF* in ruminants such as goat and cattle.

## Conclusion

In this study, the *NENF* gene sequence of goats containing intact CDS regions was cloned. Physicochemical analysis of the protein showed that it was a a hydrophilic unstable protein. After the overexpression of *NENF* in goats, it was comprehensively demonstrated by morphology and molecular biology that overexpression positively regulates the differentiation of adipocytes.

## Supplementary Material

Supplemental Material
