# Supplementary figures and images for: Neuron-derived neurotrophic factor promotes the differentiation of intramuscular and subcutaneous adipocytes in goat
Source: Anim Biotechnol. 2024 May 13;35(1):2346223. doi: 10.1080/10495398.2024.2346223 (PMC12674300; doi:10.1080/10495398.2024.2346223)

**Supplementary**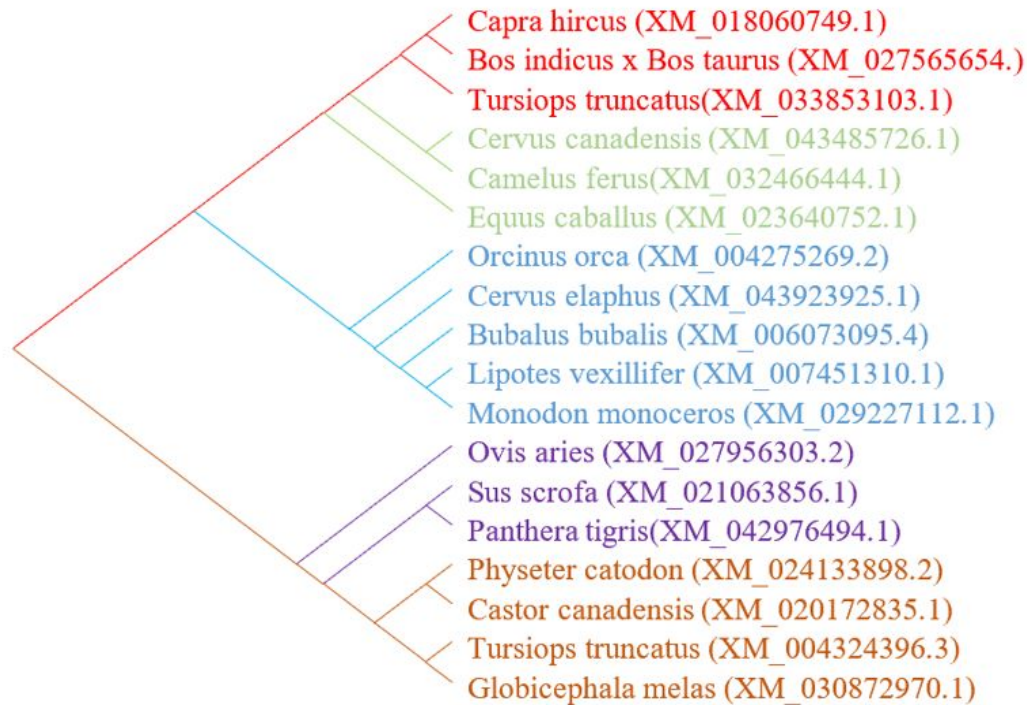**Figure S1.** Phylogenetic tree of NENF protein sequences.

Supplement: Supplemental Material [file LABT_A_2346223_SM6221.pdf]
